# A research methodology study to map the process of initiating and operating a randomised controlled trial of podoconiosis treatment in Northern Ethiopia

**DOI:** 10.1186/1745-6215-14-S1-O31

**Published:** 2013-11-29

**Authors:** Trudie Lang, Mike Clarke, Melanie Newport, Fikre Enquoselassie, Francois van Loggerenberg, Sam Franzen, Tamzin Furtado, Patricia Njuguna, Greg Fegan, Gail Davey

**Affiliations:** 1 University of Oxford, Oxford, UK; 2 Queens University, Belfast, UK; 3 Brighton and Sussex Medical School, Brighton, UK; 4 Kemri-Wellcome Programme, Kilifi, Kenya; 5 School of Public Health, Addis Ababa, Ethiopia; 6 University of Sussex, Brighton, UK

## 

There is global inequity in medical research both in terms of populations who do not have access to the best evidence-led health care and in medical and scientific staff taking part in research studies. Alongside this is international recognition that trials have become overly cumbersome in their operation and regulation and that they are too expensive. There is widespread agreement that there needs to be changes in the way trials and designed and operated in order to make them more pragmatic and thereby attractive and viable for would be researchers (and their funders). A common compliant is the lack of published examples and best practices in operational approaches in running clinical trials. Here we report our on-going research methodology study that follows the steps and processes that are involved in setting up a highly pragmatic randomised clinical trial in Ethiopia and create a process map to report how the study was initiated and then subsequently operates. The primary object of this study is to determine all the issues and challenges that the trial team faces. Secondary objectives include an analysis of the study metrics, experience data from the study personnel and whether any lessons or solutions can either be shared or turned into a tool that others can benefit from for future studies.

